# Nivolumab plus ipilimumab in metastatic melanoma: a critical appraisal focused on specific subpopulations

**DOI:** 10.3389/fonc.2023.1187840

**Published:** 2023-06-19

**Authors:** Lucía Vázquez-Montero, María del Carmen Álamo de la Gala, Luis de la Cruz-Merino

**Affiliations:** ^1^ Clinical Oncology Department, University Hospital Virgen Macarena, Seville, Spain; ^2^ School of Medicine. University of Seville, Seville, Spain; ^3^ Cancer Immunotherapy, Department of Oncohematology and Genetics, Biomedicine Institute of Seville (IBIS)/CSIC Spanish National Research Council, Seville, Spain

**Keywords:** nivolumab, ipilimumab, advanced melanoma, safety, efficacy

## Abstract

The development of immune checkpoint inhibitors has revolutionized the landscape of treatment of advanced melanoma in recent years. Based on the efficacy results of the phase III CheckMate 067 trial, nivolumab in combination with ipilimumab is one of the first-line standard options for advanced melanoma along with pembrolizumab, nivolumab, and, recently, nivolumab plus relatlimab. Counterbalancing its efficacy, nivolumab plus ipilimumab is associated with severe immune-related toxicity. This article will review the efficacy and safety of the nivolumab plus ipilimumab combination in advanced melanoma across phase I, II, and III clinical trials that evaluated this approach. We also explore the benefit of the combination schedule across different subgroups of patients and possible predictive biomarkers for efficacy outcomes in order to elucidate which patients could be the best candidates for combination or single-agent therapy. Patients with *BRAF*-mutant tumours, asymptomatic brain metastases, or PD-L1-negative status appear to reach better survival outcomes with the combination relative to single-agent immunotherapy.

## 1 Introduction

Melanoma is the fifth most common malign neoplasm in Western countries (1, 2). Specifically in Spain, it is estimated that there was an incidence of 7,474 new cases of melanoma in 2022 (3). Traditionally, survival has been extremely poor in the metastatic setting. New therapeutic options through targeted therapies and modern immunotherapy based on immune checkpoint inhibitors (ICIs) have dramatically changed the therapeutic scenario for this disease. Moreover, these new approaches introduce important new questions and challenges that are worth to be addressed and elucidated to offer the optimum treatment to every single patient, in a personalized approach, if possible (4, 5). The development of ICIs, which are designed to activate and boost the immune system of the host against the tumour, has transformed the treatment landscape of metastatic melanoma and significantly improved the clinical outcomes with an impact on long-term overall survival. The best-characterized checkpoint blockades possess the ability to bind to either cytotoxic T-lymphocyte associated protein-4 (CTLA-4) or programmed death receptor 1 (PD-1), which are immunosuppressive molecules expressed on T cells and other immune cells (6–8). This antibody binding to either PD-1 (nivolumab and pembrolizumab) or CTLA-4 (ipilimumab) results in the abolition of signalling in response to these inhibitory receptors’ ligands and intensifies the effector T-cell recruitment and activation in the tumour microenvironment (9).

Anti-CTLA-4 antibody ipilimumab was the first drug to show a significant improvement in overall survival in the treatment of advanced melanoma, and its use in this setting was approved in 2011 by the Food and Drug Administration (FDA) (10, 11). Subsequently, nivolumab and pembrolizumab, both targeting PD-1, have shown superior long-term efficacy and safety outcomes in comparison to ipilimumab in various phase III trials in advanced melanoma (12, 13). Therefore, ipilimumab, nivolumab, and pembrolizumab are licensed for use as monotherapies for the treatment of advanced melanoma, with the consideration of the two anti-PD1 agents as the standard of care in many centres around the world (14, 15). As PD-1 and CTLA-4 are coinhibitory molecules with a non-redundant mechanism, it was suggested that dual immunotherapy could synergize and enhance the efficacy of these treatments. This was the rationale to explore the efficacy of adding ipilimumab to nivolumab in this setting, specifically addressed in the landmark CheckMate 067 clinical trial (12).

As we will discuss further in this review, a combination of ipilimumab and nivolumab followed by nivolumab maintenance has been demonstrated to be superior to ipilimumab monotherapy for progression-free survival (PFS) and objective response rate (ORR) in untreated advanced melanoma. After a follow-up period of 6.5 years, the median overall survival (OS) has been reached in the three groups of treatment, and the combination regimen has also confirmed a significant OS improvement relative to ipilimumab monotherapy. Even though the median OS appears also improved with the combination approach relative to nivolumab monotherapy, the CheckMate 067 trial was not powered to show a statistical difference between the nivolumab group and the nivolumab plus ipilimumab group. This fact has been misleading to the scientific community. In addition, the high efficacy observed with this combination approach is counterbalanced by more severe immune-related toxicity. In advanced melanoma, the recommended dosing for the combination therapy is ipilimumab 3 mg/kg and nivolumab 1 mg/kg once every 3 weeks for four cycles followed by nivolumab maintenance 3 mg/kg every 2 weeks until progression or intolerable toxicity. This approach was FDA-approved for advanced or unresectable melanoma regardless of *BRAF* status in 2016 (16). Consequently, nivolumab plus ipilimumab has become one of the preferred options in the international melanoma guidelines along with nivolumab and pembrolizumab. Nevertheless, certain subgroups of patients seem to reach better clinical outcomes with combination therapy than others. In this sense, patients with *BRAF*-mutant tumours, brain metastases, or PD-L1-negative status appear to obtain a greater benefit with nivolumab–ipilimumab versus single-agent immunotherapy.

This article will review the efficacy and safety of the nivolumab plus ipilimumab combination in advanced melanoma across the respective phase I, II, and III clinical trials that evaluated this approach. We will also discuss the benefit of the combination regimen across different subgroups and some possible predictive biomarkers that could guide the therapeutic decisions of clinicians in daily practice.

## 2 Results of clinical efficacy and safety

### 2.1 Early studies, phase I trial

The efficacy of the combination of nivolumab and ipilimumab was firstly reported in the phase I dose-finding study (CheckMate 004) (17). In this trial, 53 patients diagnosed with stage IV or unresectable stage III melanoma received a combination regimen with ipilimumab and nivolumab at escalating doses every 3 weeks for four doses followed by nivolumab alone every 3 weeks for four cycles (concurrent regimen).

Among the 53 patients in the concurrent regimen, a total of 38% of patients received systemic therapy previously. Most patients had M1c disease (57%), none had brain metastases, and more than 30% of patients had an elevated level of lactate dehydrogenase (LDH) (38%). PD-L1 status was established by immunohistochemical testing. With PD-L1 positivity defined as expression in less than 5% of tumour cells, 38% of biopsy specimens were PD-L1 positive.

At a median follow-up of 2.5 years, an ORR of 40% was observed in the concurrent-regimen cohorts across all dose levels. A total of 16 patients (31%) had tumour reduction of 80% or more at 12 weeks, including 5 with a complete response. Objective responses were observed in both PD-L1-positive and PD-L1-negative patients without significant differences between both subgroups. Updated OS analyses were reported for patients who received combination therapy with additional 10 months of follow-up (18). In this report, the 2-year OS rate was 75% across all doses. Responses were observed regardless of *BRAF* mutational status. **Table 1** summarizes efficacy data of phase I, II, and III trials that evaluated the nivolumab and ipilimumab combination regimen.

**Table 1:** Summary of efficacy data in phase I, II, and III trials evaluating nivolumab and ipilimumab combination.

Study	Trial phase	Population	Medication	ORR	OS	Median PFS
Wolchok et al. (2013). *CheckMate 004* (17)	Phase I	N = 53 stage IV or unresectable stage III melanoma *(pre-treated: 38%)* PD-L1 ≥ 5%: 38%	NIVO 1 mg/kg + IPI 3 mg/kg	40% (CR: 9%)	2-year OS rate: 75%	None reported
Hodi et al. (2016). *CheckMate 069* (19, 20)	Phase II	N = 142 stage IV or unresectable untreated stage III melanoma BRAFm: 23% PD-L1 ≥ 5%: 30%	NIVO 1 mg/kg + IPI 3 mg/kg *vs.* IPI 3 mg/kg	59% NIVO+IPI *vs.* 11% IPI - BRAFwt: 61% *vs.* 11% - BRAFm: 52% *vs.* 10%	2-year OS rate: 63.8% NIVO+IPI *vs.* 53.6% IPI	NR *vs.* 3.0 months (HR 0.38)
Hodi et al. (2022). *CheckMate 067* (12, 21, 22)	Phase III	N = 945 stage IV or unresectable untreated stage III melanoma BRAFm: 31.5% PD-L1 ≥ 5%: 36%	NIVO 1 mg/kg + IPI 3 mg/kg *vs.* NIVO 3 mg/kg *vs.* IPI 3 mg/kg	58% NIVO+IPI *vs.* 45% NIVO *vs.* 19% IPI	7.5-year OS rate: 48% NIVO+IPI *vs.* 42% NIVO *vs.* 22% IPI	11.5 months NIVO+IPI *vs.* 6.9 months NIVO *vs.* 2.9 months IPI

BRAFm, BRAF-mutant tumours; BRAFwt, BRAF wild-type tumours; CR, complete response; IPI, ipilimumab; MEKi, MEK inhibitor; NIVO, nivolumab; NIVO+IPI, nivolumab and ipilimumab; NR, not reached; ORR, objective response rate; OS, overall survival; PD-L1, programmed death-ligand 1; PFS, progression-free survival.

Regarding safety, treatment-related adverse events (AEs) were observed in 93% of treated patients, and the most common events were rash (55%), pruritus (47%), fatigue (38%), and diarrhoea (34%). Grade 3 or 4 treatment-related AEs were noted in 53% of patients. A total of 11 patients (21%) discontinued therapy because of treatment-related AEs. The maximum tolerated dose was nivolumab 1 mg/kg and ipilimumab 3 mg/kg. Of the 17 patients treated at this dose, a considerable proportion of patients experienced a grade 3 or 4 immune-related adverse event: hepatitis (18%), diarrhoea, or colitis (12%). No treatment-related deaths were reported. **Table 2** summarizes the toxicity of nivolumab–ipilimumab regimen and monotherapies evaluated in the respective phase I, II, and III trials.

**Table 2:** Main toxicity of nivolumab–ipilimumab combination in the different phase I, II, and III trials.

Study	Trial phase	Medication	All grades	Most common (NIVO+IPI cohorts)	Grade 3–4	AEs led to discontinuation	Treatment-related deaths.
Wolchok et al. (2013). *CheckMate 004* (17)	Phase I	NIVO+IPI (at escalating doses)	93%	Rash (55%) Pruritus (47%) Fatigue (38%) Diarrhoea (34%)	53%.	21%	None
Hodi et al. (2016). *CheckMate 069* (19, 20)	Phase II	NIVO 1 mg/kg + IPI 3 mg/kg *vs.* IPI 3 mg/kg	92% NIVO+IPI *vs.* 94% IPI	Diarrhoea (45%) Fatigue (36%) Pruritus (40%)	54% NIVO+IPI *vs.* 20% IPI	30% NIVO+IPI *vs.* 9% IPI	3 NIVO+IPI *vs.* 0 IPI
Hodi et al. (2022). *CheckMate 067* (12, 21, 22)	Phase III	NIVO 1 mg/kg + IPI 3 mg/kg *vs.* NIVO 3 mg/kg *vs.* IPI 3 mg/kg	96% NIVO+IPI *vs.* 87% NIVO *vs.* 86% IPI	Diarrhoea (46%) Fatigue (38%) Pruritus (36%)	59% NIVO+IPI *vs.* 24% NIVO *vs.* 28% IPI	42% NIVO+IPI *vs.* 14% NIVO *vs.* 15% IPI	2 NIVO+IPI *vs.* 1 NIVO *vs.* 1 IPI

AEs, adverse events; IPI, ipilimumab; NIVO, nivolumab; NIVO+IPI, nivolumab and ipilimumab.

### 2.2 Phase II trial

Based on the promising efficacy results of this early phase study, a randomized, double-blind, phase II trial (CheckMate 069) of ipilimumab 3 mg/kg in combination with nivolumab 1 mg/kg or placebo was conducted in patients naïve of treatment (19). Patients received combination therapy or ipilimumab plus placebo for four 3-weekly cycles followed by nivolumab 3 mg/kg maintenance or placebo (if patients were allocated to ipilimumab alone arm) every 2 weeks until lack of clinical benefit or unacceptable toxicity. Patients who progressed in the ipilimumab monotherapy group had an option of receiving single-agent nivolumab at a dose of 3 mg/kg 2-weekly until further progression.

The study randomized 142 patients diagnosed with stage IV or unresectable stage III melanoma in a 2:1 manner to each treatment arm. Randomization was stratified by *BRAF* mutational status. Of the 142 enrolled patients, most had metastatic melanoma (87%), and 46% of the patients had M1c disease. Elevated LDH was observed in 25%, and 23% had a *BRAF* mutation. Four patients (3%) had a history of brain metastases, and all of them were assigned to the combination arm. Baseline characteristics were well balanced between the two groups of treatment. Patients with uveal melanoma, active brain metastases, or leptomeningeal metastases were excluded. Of those evaluable for PD-L1 status (118 patients), 30% were positive using a 5% cut-off. The primary endpoint was the ORR in *BRAF* wild-type melanomas, with OS being an exploratory endpoint.

Among patients with *BRAF* wild-type tumours, the confirmed ORR as assessed by investigators was 61% in the combination group versus 11% in the ipilimumab monotherapy group. ORRs in those with a *BRAF* mutation were similar at 52% for the combination group and 10% for the ipilimumab monotherapy group. The percentage of complete responses in the combination group was the same independent of *BRAF* status (22%).

At a median follow-up of 2 years, median PFS was superior with combination ipilimumab–nivolumab compared with ipilimumab monotherapy for both *BRAF* wild-type and mutant tumours (not reached *vs.* 4.4 months, HR 0.40, and 8.5 *vs.* 2.7 months, HR 0.38, respectively) (20). In all patients assigned to treatment (regardless of *BRAF* mutation), the 2-year OS rate was 63.8% in the combination group and 53.6% in the ipilimumab alone group. Median OS had not been reached in either group at that time (HR 0.74, 95% CI 0.43–1.26, p = 0.26). The study showed no difference in the 2-year OS rate between *BRAF*-mutant and *BRAF* wild-type tumours.

A prespecified exploratory biomarker analysis was conducted regarding PD-L1 status in the combination group. ORRs did not differ substantially between patients with PD-L1-positive tumours (58%) and those with PD-L1-negative expression (55%). The results were similar for OS (2-year OS rates: 67% *vs.* 60%, respectively).

Overall, the most common treatment-related AEs in the combination group were diarrhoea (45%), fatigue (36%), and pruritus (40%). Treatment-related grade 3–4 AEs were more common in the combination group than in the ipilimumab group (54% *vs.* 20%) and led to therapy discontinuation in 30% in the combination group and 9% in the ipilimumab group. Three deaths in the combination group were attributed to treatment-related AEs, whereas no deaths from treatment-related AEs were reported in the ipilimumab group.

### 2.3 Randomized comparative phase III trials

#### 2.3.1 CheckMate 067

CheckMate 067 was an international, randomized, double-blind, phase III study in which untreated patients with unresectable or advanced melanoma were randomly assigned to receive one of the following regimens: combination nivolumab 1 mg/kg and ipilimumab 3 mg/kg three-weekly for four cycles, ipilimumab 3 mg/kg every 3 weeks for four cycles, or nivolumab 3 mg/kg two-weekly for four cycles (12). The combination therapy was followed by nivolumab 3 mg/kg maintenance every 2 weeks and continued until lack of clinical benefit or toxicity.

Randomization was stratified by tumour PD-L1 status (positive *vs.* negative; using a 5% cut-off of tumour expression assessed utilizing the same method as previously explained), *BRAF* mutational status, and advanced metastasis stage (M0-1b *vs.* M1c). PFS and OS were coprimary endpoints. Specifically, the study was only powered to compare both nivolumab-containing arms with ipilimumab monotherapy.

Of the 945 randomized patients, most of them had M1c disease (58%), approximately a third of them had a BRAF mutation (31.5%), 23.6% were PD-L1 positive, and 36.1% had an elevated LDH level. Baseline characteristics were well balanced across the three arms.

PFS data was reported after a median follow-up time of 12 months, remaining unchanged during additional follow-up. Combination therapy significantly reduced the chance of progression or death compared with ipilimumab. In the last updated report with a minimum 7.5-year follow-up time, the median PFS in the combination therapy cohort has been 11.5 months, superior to ipilimumab at 2.9 months (HR 0.42) (21). For nivolumab monotherapy, median PFS has been 6.9 months, also superior to ipilimumab (HR 0.53). ORRs at 7.5 years were 58%, 45%, and 19% with the combination, nivolumab, and ipilimumab, respectively. The median duration of response (DOR) had not been reached at 90 months in the nivolumab and ipilimumab group, was reached with nivolumab (90.8 months), and remained at 19.2 months with ipilimumab since the report published after 3 years of follow-up. Ipilimumab in combination with nivolumab also demonstrated a significant benefit in OS relative to ipilimumab. The study, with a minimum 7.5-year follow-up, continued to demonstrate substantially improved OS with nivolumab-containing treatment arms compared with ipilimumab alone. The 7.5-year OS rates were 48%, 42%, and 22% for combination, nivolumab, and ipilimumab, respectively. Median OS was 72.1, 36.9, and 19.9 months in the combination, nivolumab, and ipilimumab groups, respectively (HR for death with nivolumab–ipilimumab *vs.* ipilimumab, 0.52; HR for death with nivolumab *vs.* ipilimumab, 0.63).

Durable clinical benefit has been observed across clinically relevant subgroups, including those based on *BRAF* mutational status or baseline M1c disease, except for those patients with a PD-L1-positive status.

Regarding *BRAF* mutational status, better survival outcomes with a nivolumab–ipilimumab regimen have been observed in patients with *BRAF*-mutant tumours. OS rates at 7.5 years were 57%, 42%, and 25% in patients with *BRAF*-mutant tumours and 43%, 41%, and 21% in patients with *BRAF* wild-type tumours in the nivolumab–ipilimumab, nivolumab, and ipilimumab treatment arms, respectively. Notably, in the PD-L1-negative subgroup, there was a larger advantage in OS with combination therapy relative to nivolumab and ipilimumab monotherapies (median 65.9 *vs.* 35.9 *vs.* 18.4 months, respectively). In the PD-L1-positive subgroup, median OS had not been reached at 77 months in the nivolumab and ipilimumab group, and it was reached in the nivolumab group (64.3 months), and both had superior median OS when compared to ipilimumab (28.9 months). The 6.5-year OS rates were 48% with nivolumab plus nivolumab, 42% with nivolumab, and 21% with ipilimumab in patients with PD-L1 < 5% and 54%, 49%, and 30% in patients with PD-L1 ≥ 5%, respectively (22).

Regarding safety, the most common AEs in the combination group were the same as those described in the phase II CheckMate 069 trial: diarrhoea (46%), fatigue (38%), and pruritus (36%). The incidence of treatment-related AEs of grade 3–4 was substantially higher in the combination arm (59%) than in the nivolumab arm (24%) or the ipilimumab arm (28%). The most frequent immune-related adverse events of grade 3 or 4 were diarrhoea (in 8% of patients in the combination group, 3% in the nivolumab group, and 4% in the ipilimumab group), colitis (in 7%, 1%, and 9%, respectively), and increased alanine aminotransferase (in 6%, 1%, and 1%, respectively). Treatment-related AEs of any grade that led to discontinuation of the study drug occurred in 42% of the patients in the combination group, 14% in the nivolumab group, and 15% in the ipilimumab group. Two deaths in the combination group were related to the study drug (one from autoimmune myocarditis and one from liver necrosis). Two more deaths related to the study drug were reported: one death due to colon perforation in the ipilimumab group and one due to neutropenia in the nivolumab group.

#### 2.3.2 Alternative nivolumab–ipilimumab combination schedules: CheckMate 511 trial

CheckMate 511 is another important phase III trial conducted to determine if lower doses of ipilimumab using a combination of nivolumab 3 mg/kg plus ipilimumab 1 mg/kg could improve the safety profile of the standard combination of nivolumab 1 mg/kg plus ipilimumab 3 mg/kg (23).

A total of 360 patients with untreated, unresectable stage III or IV melanoma were randomized to receive nivolumab 3 mg/kg plus ipilimumab 1 mg/kg or nivolumab 1 mg/kg plus ipilimumab 3 mg/kg once every 3 weeks for four cycles followed by nivolumab 480 mg maintenance every 4 weeks. The primary endpoint was focused on the comparison of treatment-related grade 3 to 5 AEs.

Incidence of treatment-related grade 3 to 5 AEs was significantly lower in the nivolumab 3 mg/kg and ipilimumab 1 mg/kg group compared with the nivolumab 1 mg/kg and ipilimumab 3 mg/kg group (33.9% *vs.* 48.3% p = 0.006). Rates of most treatment-related AEs were lower in the nivolumab 3 mg/kg and ipilimumab 1 mg/kg arm, with overall lower incidence of grade 3–4 EAs in the nivolumab 3 mg and ipilimumab 1 mg arm compared with the standard regimen arm especially related to the lower incidence of gastrointestinal (6.1% *vs.* 10.7%), hepatic (7.2% *vs.* 16.3%), and endocrine (2.8% *vs.* 7.3%) toxicity. The incidence of grade 3–4 AEs leading to discontinuation was lower in the nivolumab 3 mg/kg and ipilimumab 1 mg/kg arm when compared with the standard regimen arm (16.7% *vs.* 27.5% respectively).

The results of this study demonstrate that the safety profile of low-dose ipilimumab in combination with the approved dose of nivolumab in advanced melanoma is superior to that of the standard combination regimen.

### 2.4 Efficacy data of nivolumab and ipilimumab in subpopulations of interest

#### 2.4.1 Brain metastasis setting

With respect to patients with brain metastases, there are essentially two phase II studies performed addressing the activity of the combination. **Table 3** summarizes the main efficacy results of nivolumab and ipilimumab in patients with brain metastases, along with other clinically relevant subgroups.

**Table 3:** Main efficacy results with nivolumab and ipilimumab in clinically relevant subgroups.

Brain metastases	*BRAF*-mutant tumours	Mucosal melanoma	Elevated LDH levels population	PD-L1 negative tumours
NCT02374242 trial (24): Intracranial responses: 46% NIVO+IPI *vs.* 20%	SECOMBIT trial (25): 2-year OS rate: 65% arm A (1st line: BRAFi/MEKi) *vs.* 72% arm B (1st line: NIVO+IPI) *vs.* 69% arm (1st line: BRAFi/MEKi for 8 weeks + NIVO+IPI)	Pooled analysis CheckMate 069 + CheckMate 067 (26): PFS: 5.9 months NIVO+IPI *vs.* 3 months NIVO *vs.* 2.7 months IPI ORR: 37.1% NIVO+IPI *vs.* 23.3% NIVO *vs.* 8.3% IPI	CheckMate 067 (27): 5-year OS: • LDH > ULN: 38% NIVO+IPI *vs.* 28% NIVO *vs.* 15% IPI • LDH > 2× ULN: 28% NIVO+IPI *vs.* 14% NIVO *vs.* 7% IPI	CheckMate 069 (20): 2-year OS rates (*NIVO+IPI cohort*): 67% PD-L1 positive *vs.* 60% PD-L1 negative
CheckMate 204 (28): Intracranial responses: 57% NIVO+IPI.	DREAMseq trial (29): 2-year OS rate: 72% arm A (first line: NIVO+IPI) *vs.* 52% arm B (first line: BRAFi/MEKi)		CheckMate 069 (27): Median OS: • LDH < ULN: NR NIVO+IPI *vs.* NR IPI (HR 0.72) • LDH > ULN: 13.7 months NIVO+IPI *vs.* 7.2 months IPI (HR 0.67)	Checkmate 067 (22): 6.5-year OS rates: • PD-L1 negative: 48% NIVO+IPI *vs.* 42% NIVO *vs.* 21% IPI • PD-L1 positive: 54% NIVO+IPI *vs.* 49% NIVO *vs.* 30% IPI

BRAFi, BRAF inhibitor; IPI, ipilimumab; LDH, lactate dehydrogenase; MEKi, MEK inhibitor; NIVO, nivolumab; NIVO+IPI, nivolumab and ipilimumab; NR, not reached; ORR, objective response rate; OS, overall survival; PD-L1, programmed death-ligand 1; PFS, progression-free survival; ULN, upper limit of the normal range.

A multicentre phase II study in Australia published in 2018 showed that nivolumab combined with ipilimumab also may provide benefits in this setting (24). A total of 79 patients were enrolled in this study. Those with asymptomatic brain metastases with no previous local brain therapy were randomly assigned to receive ipilimumab 3 mg/kg and nivolumab 1 mg/kg for four cycles followed by nivolumab 3 mg/kg maintenance every 2 weeks, or nivolumab 3 mg/kg alone. Patients with brain metastases in whom local therapy had failed or those with neurological symptoms or leptomeningeal disease were allocated to a non-randomized cohort and were treated with nivolumab 3 mg/kg every 2 weeks. Intracranial responses were achieved by 46% of patients receiving combination therapy and 20% in the asymptomatic nivolumab group at a median follow-up of 17 months. Intracranial complete responses occurred in 17% of patients treated with the combination and 12% with nivolumab monotherapy. Of those who had received prior therapy, symptoms, or leptomeningeal disease, 6% achieved a response; however, none achieved a complete response.

The CheckMate 204 was a non-randomized prospective study of patients with metastatic melanoma with brain metastases, which gave additional evidence for nivolumab–ipilimumab activity in this scenario (28). A total of 94 patients with metastatic melanoma non-irradiated and asymptomatic brain metastases (tumour diameter, 0.5 to 3 cm) were enrolled. The primary endpoint was the rate of intracranial clinical benefit using the combination of nivolumab 1 mg/kg plus ipilimumab 3 mg/kg every 3 weeks for four doses followed by nivolumab 3 mg/kg maintenance every 2 weeks. The rate of intracranial clinical benefit at a medium follow-up of 14 months was 57%, with 26% of patients achieving a complete response.

In both studies, the safety data were similar to those of prior studies in patients with melanoma without brain metastases.

#### 2.4.2 *BRAF*-mutated population: sequential therapies

Another area of interest is that of *BRAF* mutation-positive tumours (also see **Table 3**). The efficacy of nivolumab–ipilimumab in this setting was first pragmatized in the SECOMBIT trial (25). This study was designed to evaluate survival outcomes for sequential treatment with immunotherapy and targeted therapy in patients with *BRAF*-mutant metastatic melanoma.

A total of 209 patients were randomized to receive encorafenib 450 mg orally once daily plus binimetinib 45 mg orally twice daily until the progression of disease followed by ipilimumab 3 mg/kg plus nivolumab 1 mg/kg every 3 weeks four cycles and then nivolumab 3 mg/kg every 2 weeks (arm A), ipilimumab plus nivolumab until progression followed by encorafenib plus binimetinib (arm B), or encorafenib plus binimetinib for 8 weeks and ipilimumab plus nivolumab until progression when they would receive encorafenib plus binimetinib (arm C). The 2-year OS rate, which was the primary endpoint, was favourable for arm B (65% in arm A *vs.* 73% in arm B *vs.* 69% in arm C).

In conclusion, this study sustained the fact that eligible patients with BRAF-mutant melanoma reach clinical benefit with combination immunotherapy as the first line of treatment.

Based on the promising data reported on the previously mentioned phase II SECOMBIT trial as well as the well-known efficacy of *BRAF/MEK* inhibitor combinations in this setting, a phase III trial was conducted to determine which treatment sequence could achieve better efficacy.

In the DREAM-seq trial, patients with untreated *BRAF*-mutant metastatic melanoma were randomly assigned to receive either a combination of nivolumab 1 mg/kg plus ipilimumab 3 mg/kg 3-weekly for four doses and then nivolumab 240 mg maintenance every 2 weeks (arm A) or dabrafenib 150 mg twice a day plus trametinib 2 mg once daily (arm B) and at disease progression were enrolled to receive the alternative therapy (29). Their 2-year OS rate was the primary endpoint. Patients were stratified by Eastern Cooperative Oncology Group (ECOG) (0 or 1) and LDH level (normal or elevated).

Of the 265 enrolled patients, most had M1c disease (60%), and elevated LDH was observed in 40% of patients, with most characteristics being well balanced between the two groups.

The study showed that the sequence starting with nivolumab and ipilimumab resulted in a 20% absolute improvement in the 2-year OS rate compared with the sequence that started with dabrafenib and trametinib (72% *vs.* 52% p = 0.010). Median PFS also favoured arm A (11.8 *vs.* 8.5 months p = 0.054). For all subsets of patients, OS was numerically better for the sequence that started with immunotherapy. Notably, even in the subgroup of those patients with “favourable” characteristics (those with ECOG 0, normal LDH, and no M1c disease), starting with nivolumab and ipilimumab showed a trend for improved OS (86.2 *vs.* 54.1 months p = 0.059). Furthermore, in patients with non-favourable characteristics, the sequence beginning with immunotherapy showed a significant improvement in OS (67.4 *vs.* 49.8 months p = 0.045).

These findings supported the results of the SECOMBIT trial, establishing nivolumab and ipilimumab followed by *BRAF* and *MEK* inhibitor combination at disease progression as the preferred treatment sequence for a large majority of patients with advanced BRAF-mutant melanoma.

#### 2.4.3 Mucosal melanoma

Mucosal melanoma is a rare melanoma subtype (accounting for 2% or less of all melanomas) that is characterized to be aggressive and resistant to traditional therapies. A major challenge with mucosal melanoma is that well-established protocols for staging and treatment are lacking (30, 31). This subtype is very poorly represented in trials evaluating anti-PD1-based regimens in advanced melanoma. The evidence of the use of ipilimumab and anti-PD-1 agents in this setting is based on small study populations, retrospective analyses, and single-case reports (32–34).

A pooled analysis of data from patients with mucosal melanoma who receive nivolumab alone or combined with ipilimumab in clinical trials was conducted to better understand the benefit of anti-PD-1-based therapy (26). To evaluate the efficacy and safety of nivolumab combined with ipilimumab in mucosal melanoma, data were pooled from CheckMate 067 and CheckMate 069 trials. Among 889 patients who received nivolumab monotherapy, 86 patients (10%) with mucosal melanoma were included. For those who received the nivolumab plus ipilimumab combination (n = 407), 35 patients (9%) with mucosal melanoma were included, and 36 of 357 patients (10%) with mucosal melanoma had received ipilimumab monotherapy. Median PFS was 3, 5.9, and 2.7 months for patients with mucosal melanoma who received nivolumab monotherapy, combination therapy, and ipilimumab monotherapy, respectively, and their ORRs were 23.3%, 37.1%, and 8.3%, respectively (26).

Although the activity of nivolumab plus ipilimumab in mucosal melanoma seems to be lower than in cutaneous melanoma, the combination regimen appears to have greater efficacy than either agent alone.

#### 2.4.4 Elevated LDH level subpopulation

Elevated LDH level is a risk factor for aggressive disease represented by approximately one-third of patients in the overall populations of the mentioned studies evaluating nivolumab plus ipilimumab in advanced melanoma. Survival outcomes in these patients are generally worse than in patients with a normal level of this biomarker. In these patients with this unfavourable characteristic, survival outcomes are numerically higher for those treated with nivolumab plus ipilimumab and nivolumab compared with ipilimumab.

In the 5-year follow-up of the CheckMate 067 trial, 5-year OS rates among patients with elevated LDH levels were 38%, 28%, and 15% in the nivolumab plus ipilimumab, nivolumab, and ipilimumab groups, respectively (27). Specifically, survival is markedly reduced in those patients who have an LDH level increased to twice the upper limit of normal. In this subgroup, 5-year OS rates were 28%, 14%, and 7% in the nivolumab plus ipilimumab, nivolumab, and ipilimumab groups, respectively (27). In CheckMate 069, the benefit of nivolumab plus ipilimumab compared to ipilimumab appears to be slightly superior in patients with elevated LDH concentrations than in patients with normal LDH levels. Among patients with normal LDH levels, median OS had not been reached in either group (HR 0.72), and among patients with elevated LDH levels, median OS was 13.7 *vs.* 7.2 months in the nivolumab plus ipilimumab *vs.* ipilimumab group, respectively (HR 0.67) (20).

Nonetheless, the efficacy data of the nivolumab plus ipilimumab combination in this population are suboptimal, and these patients generally have a negative prognosis.

## 3 Discussion

In the last few years, most of the commonly used international clinical guidelines around the globe have considered the standard of care two immunotherapeutic strategies: anti-PD1 single therapy (nivolumab or pembrolizumab) and the combination of anti-CTLA4 and anti-PD1 (ipilimumab plus nivolumab). Recently, a new combination with anti-PD1 plus anti-LAG3 (relatlimab) has been added as a new option of treatment based on the results of the RELATIVITY-047 trial (35). Its use in this setting was FDA-licensed in March 2022 and with marketing authorization by European Medicines Agency (EMA) on September 2022 (36, 37).

Long-term follow-up efficacy data from phase II and III trials and especially the toxicity burden of the anti-CTLA4 and anti-PD1 combination have raised questions on what could be the best frontline treatment for advanced melanoma and which are the best candidates for combination or single-agent therapy. The efficacy data available of nivolumab and ipilimumab in first-line treatment from two randomized trials show a substantial improvement in PFS and ORR and include the longest median OS (72.1 months) reported to date in phase III studies in patients with metastatic melanoma. Notably, this advantage has been maintained with long-term follow-up. Likewise, the combination also provides durable responses with median DOR not reached with the combination in the latest update of the phase III study discussed above after a median follow-up of 7.5 years.

Long-term survival analysis in the CheckMate 067 trial has shown substantially improved OS with nivolumab-containing regimens compared with ipilimumab alone. However, despite CheckMate 067 being designed without a formal comparison of nivolumab plus ipilimumab and nivolumab alone groups, the median OS with the combination was approximately twice as long as nivolumab alone by a descriptive analysis in the last updated report (72.1 *vs.* 36.9 months). This fact might be indicative of a meaningful survival benefit of the combination compared with nivolumab monotherapy, but these data must be taken cautiously and need to be confirmed in a trial designed with that hypothesis in mind.

The superiority of the combination was also confirmed in the phase II CheckMate 069 trial. Although 2-year OS was higher in the combination group than in the ipilimumab alone group, this difference was not statistically significant because OS was surprisingly high in the ipilimumab alone group. In the initial phase III trial of ipilimumab in pretreated patients with unresectable or metastatic melanoma, OS with ipilimumab monotherapy at 2 years was only 25.3% (9). This difference might be justified by the fact that 70% of patients randomly assigned to ipilimumab monotherapy in the CheckMate 069 trial received subsequent treatment following disease progression, some of which were probably not commercially available during previous ipilimumab trials. The most common subsequent treatment was anti-PD-1 therapy, which was received by 29 (62%) of 47 patients assigned to the ipilimumab alone group (26 received crossover nivolumab as per protocol-prespecified, and three patients received off-study nivolumab or pembrolizumab) (19).

Analysis of subgroups from the different randomized trials appears to suggest that patients with BRAF-mutant melanoma, elevated LDH, mucosal melanoma, asymptomatic brain metastases, or PD-L1-negative status may obtain a greater benefit from the combination than from single-agent ipilimumab or anti-PD-1 monotherapies.

Regarding patients with BRAF-mutant tumours, the CheckMate 069 trial showed that nivolumab plus ipilimumab is an effective first-line treatment option regardless of BRAF mutational status. Notably, most patients in this study (77%) had BRAF wild-type melanoma, and the trial statistics focused on this population. Survival outcomes were similar for 23% of patients with BRAF-mutant melanoma, with no difference in 2-year OS rates between BRAF-mutant and BRAF wild-type tumours. However, either the small number of patients in the BRAF-mutant melanoma subgroup (even though patients were stratified by BRAF mutational status) or the fact that more ipilimumab-assigned patients received subsequent therapy (including anti-PD1 therapies and BRAF and MEK inhibitors) may limit the interpretation of these findings. Long-term efficacy data of combination nivolumab plus ipilimumab in the BRAF-mutant melanoma subgroup in the CheckMate 067 study are of much interest. Long-term follow-up confirms the trend of separation between the combination and nivolumab group curves in patients with BRAF-mutant tumours, not seen in patients with BRAF wild-type tumours. Nonetheless, the study was not powered to compare these treatment groups. The 7.5-year OS rate of 57% in patients with BRAF-mutant tumours in the nivolumab plus ipilimumab group demonstrates the efficacy of this approach in this population and highlights the question of which might represent the optimal treatment sequencing for these patients. This question was also pragmatized with the OS results of mentioned SECOMBIT and DREAMseq trials. Preliminary data available from the recently published DREAMseq trial seem to allocate nivolumab plus ipilimumab combination as the first treatment option over combination BRAF/MEK inhibitors, although these data must be confirmed with a longer follow-up period.

Regarding brain metastasis setting, patients with asymptomatic low-volume brain metastases, who have poor outcomes with immunotherapies in monotherapy, may largely benefit from the nivolumab plus ipilimumab combination. Previously mentioned trials performed in this population show evidence of high activity of the combination on brain metastases and suggest survival outcomes far above the expected for these patients. Nevertheless, it should be noted that these populations are highly selected with respect to brain metastases, and the available evidence in this setting is limited and derived from small phase II trials.

Concerning PD-L1 status, long-term follow-up of the CheckMate 067 trial shows that patients with PD-L1 expression >5% achieve superior OS rates with nivolumab plus ipilimumab compared with nivolumab and ipilimumab alone, being greater than the OS rates achieved with nivolumab plus ipilimumab, nivolumab, and ipilimumab in those patients with PD-L1 expression <5%. However, the magnitude of OS benefit achieved with nivolumab plus ipilimumab relative to nivolumab and ipilimumab alone appears to be slightly superior in the subgroup of patients with PD-L1 expression <5% (HR 0.52 for nivolumab–ipilimumab *vs.* ipilimumab in PD-L1 < 5% (95% CI 0.40–0.66) *vs.* HR 0.57 for nivolumab–ipilimumab *vs.* ipilimumab in PD-L1 > 5% (95% CI 0.37–0.90)) (24). The comparison of nivolumab plus ipilimumab and nivolumab groups was performed by a descriptive analysis (HR 0.83 for nivolumab–ipilimumab *vs.* nivolumab in PD-L1 < 5% (95% CI 0.64–1.08) *vs.* HR 0.93 for nivolumab–ipilimumab *vs.* nivolumab in PD-L1 > 5% (95% CI 0.58–1.49)). This difference becomes more evident if survival outcomes are compared using a PD-L1 1% cut-off. The 6.5-year OS rates were 48% with nivolumab plus ipilimumab, 35% with nivolumab, and 21% with ipilimumab in patients with PD-L1 < 1% tumours, and 51% with nivolumab plus ipilimumab, 50% with nivolumab, and 25% with ipilimumab in patients with PD-L1 > 1% tumours, respectively (13). Nevertheless, the expression level of PD-L1 used as cut-off to perform the statistical analysis by subgroups in the pivotal trials was 5%.

In this respect, PD-L1 cannot be considered strictly an optimum biomarker to discriminate the benefit of nivolumab–ipilimumab relative to monotherapies in terms of overall survival, although it can be considered with other factors previously to establish the first-line treatment in most of the cases.

Regarding the safety profiles of both treatments (nivolumab plus ipilimumab and nivolumab monotherapy), the incidence of serious treatment-related AEs and the incidence of discontinuations related to toxicity favours nivolumab monotherapy over nivolumab plus ipilimumab combination. Specifically, the incidence of treatment discontinuations in the combination group is considered very high, suggesting that this treatment has low tolerability. Notably, the population included in the mentioned randomized clinical trials was younger with a better ECOG status than the population who was enrolled in the pivotal trial that assessed nivolumab in monotherapy for advanced melanoma (CheckMate 066) (38).

To conclude, the combination of nivolumab and ipilimumab suggests a benefit over nivolumab monotherapy in terms of PFS and ORR and non-significance in OS in the overall study population, although it must be always considered that the CheckMate 067 trial was not designed for comparison of these two treatments groups. Nivolumab plus ipilimumab may have a superior benefit in patients with asymptomatic brain metastases and patients with BRAF-mutant tumours. Although PD-L1 is not a reliable biomarker to determine a clear impact on overall survival, it could be useful in certain circumstances to support the indication of the combination versus single therapy, together with other factors previously discussed. It is worth reminding that nivolumab plus ipilimumab is not a harmless therapy, and it is necessary to individualize its use in every patient considering the toxicity profile of the combination.

## Author contributions

LV-M and MG: These authors contributed equally to this work and share first authorship. MG and LC-M: These authors share senior authorship. All authors contributed to the article and approved the submitted version.
